# Draft Genome Sequence of *Sphingobacterium* sp. CZ-UAM, Isolated from a Methanotrophic Consortium

**DOI:** 10.1128/genomeA.00792-17

**Published:** 2017-08-17

**Authors:** José Luis Steffani-Vallejo, Cristal Zuñiga, Pablo Cruz-Morales, Luis Lozano, Marcia Morales, Cuauhtemoc Licona-Cassani, Sergio Revah, José Utrilla

**Affiliations:** a StrainBiotech, Parque de Innovación AGROBIOTEC, Irapuato, Guanajuato, Mexico; b Departamento de Procesos y Tecnología, Universidad Autónoma Metropolitana-Cuajimalpa, Ciudad de México, Mexico; c Department of Pediatrics, University of California, San Diego, La Jolla, California, USA; d Centro de Ciencias Genómicas, Universidad Nacional Autónoma de México, Cuernavaca, Morelos, Mexico; e Centro de Biotecnología-FEMSA, Tecnológico de Monterrey, Monterrey, Nuevo León, Mexico

## Abstract

*Sphingobacterium* sp. CZ-UAM was isolated from a methanotrophic consortium in mineral medium using methane as the only carbon source. A draft genome of 5.84 Mb with a 40.77% G+C content is reported here. This genome sequence will allow the investigation of potential methanotrophy in this isolated strain.

## GENOME ANNOUNCEMENT

Methane (CH_4_) is produced by natural and human-related activities; it is one of the main greenhouse gases, with a 25-fold greater global warming potential than CO_2_ (1). CH_4_-consuming bacteria are known as methanotrophs, and they can grow using CH_4_ as their only carbon source. Biological treatments are the best option to eliminate CH_4_ as an atmospheric contaminant when its percentage is below the explosive limit (5% in air) (2, 3). There are many sources of cheap and diluted CH_4_ that can be used as feedstock (4). Therefore, it is environmentally urgent to study new methanotrophs with higher CH_4_ uptake rates and to explore their metabolic potential to convert CH_4_ into added-value products (3, 5, 6). Here, we report the genome sequence of the novel potential methanotroph *Sphingobacterium* sp. CZ-UAM, which was isolated from a methanotrophic consortium from a wastewater treatment plant (7). The isolated strain is able to grow on mineral medium with a 5% CH_4_ atmosphere as its sole carbon source.

Genomic DNA from *Sphingobacterium* sp. CZ-UAM was extracted by an in-house phenol-chloroform protocol, RNase treated, and gel verified for quality. Then, a paired-end (average size, 527 bp) genomic DNA library was generated using commercial kits according to the manufacturers’ instructions (TruSeq Nano, Illumina, San Diego, CA, USA). The DNA library quality was verified using a 2100 BioAnalyzer (Agilent Genomics, Santa Clara, CA, USA). The genome sequencing was performed using an Illumina HiSeq 2500 system through the standard rapid sequencing protocol for cluster generation and sequencing by synthesis, which resulted in a total of 3,891,197 reads for a depth coverage of 300×. Genome assembly was performed using SPAdes version 3.9.0 (8) and IDBA_UD version 1.1.1 (9). Then, SSPACE version 3.0 (10) and Metassembler version 1.5 (11) were used for merging and scaffolding the obtained contigs. After genome assembly and scaffolding, 12 contigs were obtained. Phylogenetic analysis of the 16S rRNA was performed by extracting the previously annotated 16S ribosomal gene sequence, as well as the annotated *Sphingobacterium* sequences in the NCBI database. All the sequences were aligned using Muscle software (12) and edited using Gblocks (13) prior to phylogenetic tree construction using Bayesian inference. The analyses indicate that the isolated strain is a member of the *Sphingobacterium* genus. The analysis of the full 16S rRNA gene sequence with the EZBioCloud database indicates higher identity with *S. pakistanence* (97.1%) and *S. canadense* (96.8%), *S. ginsenosidimutans* (96.46%), and *S. detergens* (95.39%) (14). Of the few available *Sphingobacterium* sequencing projects, none of them have been reported as having the potential to be a methanotroph. The availability of the *Sphingobacterium* sp. CZ-UAM genome will contribute to the study of the *Sphingobacterium* genus and will provide information on the molecular basis of this strain’s ability to consume CH_4_ as a sole carbon source.

### Accession number(s).

This whole-genome shotgun project has been deposited at DDBJ/ENA/GenBank under the accession no. MTCM00000000. The version described in this paper is the first version, MTCM01000000 (BioSample SAMN06215933).
